# New York State Veterinary Legislation

**Published:** 1896-02

**Authors:** 


### LEGISLATION.

### NEW YORK STATE VETERINARY LEGISLATION.

THE following Act, which asks for an extension of the time of registering for non-graduates and graduates who have been too ignorant, careless, or negligent to register, was offered by Mr. C. C. Cole in the Assembly at Albany. We are informed that it is instigated by certain graduates who have made cat's-paws of non-graduates to save themselves. Its object in Section 1 is to obtain extension for time of registration. The object of Section 2 in changing the number of other clauses we have not been able to solve :

Section 1. Section one hundred and seventy-one of article ten of the public health law, being chapter six hundred and sixty-one of the laws of eighteen hundred and ninety-three, as amended by chapter eight hundred and sixty of the laws of eighteen hundred and ninety-five, is hereby amended to read as follows:

§ 171. Qualifications for practice: No person shall practice veterinary medicine after September first, eighteen hundred and ninety-six, unless previously registered and legally authorized, unless licenced by the regents and registered as required by this article; nor shall any person practice veterinary medicine who has ever been convicted of a felony by any court, or whose authority to practice is suspended or revoked by the regents on recommendations of a State board.

§ 2. Sections one hundred and eighty, one hundred and eighty-one, one hundred and eighty-two, one hundred and eighty-three, and one hundred and eighty-four of article ten of the public health law, as added by such chapter eight hundred and sixty of the laws of eighteen hundred and ninety-five, are hereby renumbered, and shall hereafter be designated respectively as sections one hundred and seventy-nine-a, one hundred and seventy-nine-b, one hundred and seventy-nine-c, one hundred and seventy-nine-d, and one hundred and seventy-nine.

§ 3. This Act shall take effect immediately.

Active work was at once undertaken by the officers of the New York State Veterinary Medical Society, and by Dr. O'Shea, Chairman of the Legislative Committee of the Veterinary Medical Association of New York County, to defeat this amendment. Dr. O'Shea issued the following letter to several hundred veterinarians of the city and State of New York :

NEW YORK, January 27, 1896.

DEAR SIR: On behalf of the Committee on Legislation of our Association, I beg to call your attention to Bill No. 249, introduced in the Assembly by Mr. C. C. Cole, seeking to amend the Public Health Law relating to the practice of veterinary medicine by extending the time for registry until the first of September, 1896.

You know how hard veterinary surgeons in New York have fought such endeavors in the past, both singly and through the Society, and you will readily perceive that all our efforts will come to naught unless we all array ourselves again against this proposed legislation, that will once more open the doors of our profession to a lot of unqualified applicants.

In addition to the action that will be taken by our Committee before the Legislature, we deem it wise that all practising veterinary surgeons in New York City should address individual letters of protest to Hon. H. T. Andrews, Chairman of the Committee on Public Health of the Assembly, before whom the matter will come up on Wednesday at 2 P.M. As the time is short, we suggest that you address him immediately.

Yours truly, ATHUR O'SHEA,
Chairman of the Committee on Legislation.

The following is a sample of the argument submitted to the Legislative Committee:

NEW YORK, January 28, 1896.

THE HON. H. T. ANDREWS, Chairman of the Committee on Public Health,
Assembly Chambers, Albany, N. Y.

SIR: We have the honor to ask of your Committee an adverse report upon No. 249, Int. 244, in Assembly: An Act introduced by Mr. C. C. Cole January 16th, for the following reasons: In 1886 a satisfactory law was passed for the improvement of the status of the veterinarian and a gurantee to the agriculturist of a certain degree of education and knowledge in the veterinarian. Extensions of the time for registration were made each year until 1893, when an extension bill was vetoed. Every practitioner in the State was notified time and again of the law and failed to comply with it. Now these practitioners who ask for an extension are either so ignorant that they could not read the veterinary journals and the circulars sent them asking them to comply with the law within the required time, or if they have been criminal in knowing the law and practising without being registered at any time since 1886. For the most complete argument I would refer you to Governor Flower's veto of an extension act in 1893. This law to be amended went into effect last year, and certain good citizens have gone to the expense and labor of complying with its requirements, and now the negligent, and those who have been *illegally* practising within the last year, ask for a personal favor against the educational interest of the community.

The veterinary colleges of the State of New York have complied with the law of 1895, Chapter 860 by requiring the Regents' preliminary examination, and have thereby sacrificed incomes by having a smaller number of students, but are gratified in the result of having more intelligent students.

Very respectfully, your obedient servant,
R. S. HUIDEKOPER,
President of the Veterinary Medical Association of New York County.

One of the examiners of the State Board offered as a substitute an extension bill as follows, to include graduates only which may be submitted:

§ 1. Laws of 1895, Ch. 860, § 179, is hereby amended by adding the following:

Any graduate of a registered veterinary school who received his degree prior to July 1, 1895, and has practised veterinary medicine in some county in New York State, but who failed to register in the Veterinary Medical Register of the county in which he so practised, may, on unanimous recommendation of the State Board of Veterinary Medical Examiners, receive from the Regents a certificate which will entitle him to register as a veterinary practitioner in the county of his residence, or practice at any time before September 1, 1896.

§ 2. This Act shall take effect immediately.

This amendment was opposed by one of the Board, Dr. Huidekoper, as explained in his letter to the Board of Examiners, which follows:

NEW YORK, January 28, 1896.

BOARD OF VETERINARY MEDICAL EXAMINERS, STATE OF NEW YORK.

DEAR DOCTOR: In reply to my last letter to each of the examiners, I have received Dr. Law's letter, January 27th, Dr. Kelly's, January 27th, and Dr. Hinkley's letter, January 26th, and you have my acquiescence to the substitution of the amendment which was sent me to be made in place of the Cole Bill, which we expect to defeat; but I give this acquiescence with the distinct understanding that on the first day of September, 1896, we shall have raised a fund and employed proper counsel to prosecute every offender, and find out whether the so-called laws of 1895 and the present amendment is law or not.

I, for one, will have nothing more to do with this easy-going accommodation. We evaded the strict enforcement of the law in certain cases; we then picked out a Dr. Comstock in Albany, and he, like a man, passed an examination, and a very good one, and earned the credit of being the first man to comply with the law.

Now, we propose to accommodate a lot of loafers around the country who are too ignorant to read the journals, and know the law requirements since 1886, or, if they did know the law, were too poor citizens to comply with the law. I think this explains distinctly my feelings.

Under protest I acquiesce, and authorize you to sign my name after the signatures of the other four examiners are attached.

Very truly yours, RUSH S. HUIDEKOPER.

The Veterinary Legislative Committee of the Association has also introduced a bill to rectify the error of the legal counsel who freed the veterinarian of country counties from jury duty, but left those of New York City and Brooklyn subject to duty. This will undoubtedly pass easily, but by now becoming "special" legislation will require the approval of the Mayor of the city, as well as that of the Governor of the State.

